# Activity of the Human Rhinovirus 3C Protease Studied in Various Buffers, Additives and Detergents Solutions for Recombinant Protein Production

**DOI:** 10.1371/journal.pone.0153436

**Published:** 2016-04-19

**Authors:** Raheem Ullah, Majid Ali Shah, Soban Tufail, Fouzia Ismat, Muhammad Imran, Mazhar Iqbal, Osman Mirza, Moazur Rhaman

**Affiliations:** 1 Drug Discovery and Structural Biology group, Health Biotechnology Division, National Institute for Biotechnology and Genetic Engineering (NIBGE), Faisalabad, Pakistan; 2 Pakistan Institute of Engineering and Applied Sciences, P.O. Nilore, Islamabad, Pakistan; 3 Department of Drug Design and Pharmacology, Faculty of Health and Medical Sciences, University of Copenhagen, Copenhagen, Denmark; Centro Nacional de Biotecnologia - CSIC / CIF Q2818002D, SPAIN

## Abstract

Proteases are widely used to remove affinity and solubility tags from recombinant proteins to avoid potential interference of these tags with the structure and function of the fusion partner. In recent years, great interest has been seen in use of the human rhinovirus 3C protease owing to its stringent sequence specificity and enhanced activity. Like other proteases, activity of the human rhinovirus 3C protease can be affected in part by the buffer components and additives that are generally employed for purification and stabilization of proteins, hence, necessitate their removal by tedious and time-consuming procedures before proteolysis can occur. To address this issue, we examined the effect of elution buffers used for common affinity based purifications, salt ions, stability/solubility and reducing agents, and detergents on the activity of the human rhinovirus 3C protease using three different fusion proteins at 4°C, a temperature of choice for purification of many proteins. The results show that the human rhinovirus 3C protease performs better at 4°C than the frequently used tobacco etch virus protease and its activity was insensitive to most of the experimental conditions tested. Though number of fusion proteins tested is limited, we expect that these finding will facilitate the use of the human rhinovirus 3C protease in recombinant protein production for pharmaceutical and biotechnological applications.

## Introduction

Affinity purification and solubility enhancement tags are essential tools that aid in the overexpression and downstream processing of recombinant proteins [1, 2]. Some tags do not alter the structural and functional integrity of the target protein; however, a large number do and it is essential to remove them by proteolytic cleavage. Thus, in certain cases, the choice of tags and the design of the recognition sites for proteases that will remove them become critical though cleavage of the protease site could largely depends on the nature of fusion protein [3]. Specific and nonspecific proteases are used to remove tags. Proteases with specific recognition sites include human rhinovirus 3C protease (HRV 3C), tobacco etch virus (TEV) protease, factor Xa, thrombin, enterokinase and SUMOstar protease [3, 4]. TEV protease is a 27 kDa catalytic domain of the nuclear inclusion-a protease. It cuts efficiently between glutamine and glycine in the canonical recognition site ENLYFQ/G. Several features contributed to the widespread use of TEV protease: (1) stringent cleavage-sequence specificity, (2) post-cleavage release of the target protein with, at the most, an added G to the N-terminus [5], (3) rapid in-house production and (4) optimal activity in a variety of buffers and detergent solutions [4, 5]. A second well-known protease with similar cleavage stringency is the HRV 3C protease or PreScission^™^ protease. HRV 3C protease cleaves between glutamine and glycine of the canonical site LEVLFQ/GP [1, 4, 6, 7]. To our, knowledge no systematic study has been reported on the activity of HRV 3C protease under different experimental conditions; however, it has been suggested that the activity of HRV 3C protease is comparable to that of TEV protease [8] and that it has an increased activity 4°C [1, 2]. Despite an exhaustive literature search, we could not find a comparative study on the temperature-dependent activity of these enzymes.

Finding the appropriate buffer composition and additives (salts, sorbitol, trehalose, glycerol, arginine, proline, reducing agents and detergents) is key for the extraction and the purification of natural and recombinant proteins [9–11] and to increase their stability and solubility [9, 10, 12]. The composition of the buffer used, including any required additives, may affect the protease activity, either by a direct inhibition of the protease or by blocking the cleavage site [13]. A buffer exchange for a standard protease buffer may provide a solution; however, such procedures can be tedious, time consuming and may affect the stability of the target protein. Thus, there is great interest in proteases that are active in a wide variety of buffer solutions and temperatures to enable their exploitation for the cleavage of many recombinant proteins from their tags. Here, we have compared the activity of the HRV 3C protease to the more widely used TEV protease against a set of three fusion proteins., i) a soluble domain of a human metal-ion transport protein (designated as MTD), ii) a beta C1 protein encoded by betasatellites associated with begomoviruses and iii) a 100K protein of fowl adenovirus. These proteins were selected owing to their different nature and high production yield. Moreover, we have studied the effect of frequently used additives on the activity of the HRV 3C protease at 4°C, the temperature of choice for handling most recombinant proteins. Though number of fusion proteins tested is limited, we anticipate that the obtained results will facilitate the use of the HRV 3C protease for the cleavage of fusion tags in the production of recombinant proteins.

## Materials and Methods

### Construction of TEV and HRV 3C protease cleavable clones

HRV 3C protease cleavable clone was created by modifying pKLD116 (pKLD116-V2-TEV; generated using primers V2-TEV-F/V2-TEV-R listed in Table 1) [14, 15], which contains a His_8_-tag, an extended linker (MSTLESSGAASG), maltose binding protein (MBP) and a TEV protease cleavable site upstream from the multiple cloning site. The nucleotide sequence that encodes for the TEV protease cleavable site (ENLYFQ/G) was replaced in pKLD116-V2-TEV with the sequence of the HRV 3C protease cleavable site (LEVLFQ/GP) using the primers HRV-3C-F and HRV-3C-R to generate the construct pKLD116-V2-HRV 3C (Table 1). Using pKLD116-V2-TEV and pKLD116-V2-HRV 3C vectors, protease cleavable expression constructs for a 10 kDa soluble domain of a human metal-ion transport protein (UniprotKB Q8IWU4) (designated as MTD), a 14 kDa beta C1 protein (Q91F52) encoded by betasatellites associated with begomoviruses were created using respective primers and restriction enzymes, described in Table 1. For a 89 kDa 100K protein (E1Y6P4) of fowl adenovirus, protease cleavable clones were generated by inserting the coding sequence of 100K into the multiple cloning site of pET28a-HRV 3C and pET28a-TEV, respectively (Table 1), in reading with 5’ His_8_-HRV 3C or His_8_-TEV sequences. All cloning steps were performed in the *Escherichia coli* strain OmniMAX^™^ 2T1 (Invitrogen) and constructs were confirmed by DNA sequencing.

**Table 1:** List of oligonucleotides used for cloning.

Vector name	Primer Name	Primer sequence
**pKLD116-V2-TEV**	V2-TEV-F	5’-CACCATATGTCCACGCTGGAAAGCAGCGGCGCGGCGTCC-3’
	V2-TEV-R	5’TTCCAGCGTGGACATATGGTGATGGTGGTGATGGTGATG-3’
**pKLD116-V2-HRV 3C**	HRV-3C-F	5’-CTGGAAGTTCTGTTCCAGGGTCCGGCAGGCCTTAGCAGGTGC-3’
	HRV-3C-R	5’-GCCGGACCCTGGAACAGAACTTCCAGACTAGTGGTTGCACC-3’
**pKLD116-V2-HRV 3C- MTD (or) pKLD116-V2-TEV-MTD**	MTD-F	5’-CCAAGGCCTGAAGAGCCTGAATTACAGTGGTGTGA-3’ *Stu* I
	MTD-R	5’-CATGCCATGGTTAGTCACAGGGGTCTTCACAG-3’ *Nco* I
**pKLD116-V2-HRV 3C-betaC1 (or) pKLD116-V2-TEV-betaC1**	betaC1-F	5’-ACGA AGGCCT GATGACACCGAGCGGAACAAAC-3’ *Stu* I
	betaC1-R	5’-GCTA GCTAGC TTAAACGGTGAACTTTTTATTG-3’ *Nhe* I
**pET28a-HRV 3C-100K (or) pET28a-TEV-100K**	100K-F	5’-CAATTCCATATGGAAAGCACCGCCGA-3’ *Nde* I
	100K-R	5- GCCGGAATTCTCAGGTCGACCATTCTCTGGGC-3´ *Eco* RI

The underlined nucleotides in the primer sequences represent introduced nuclease restriction sites, and the overhangs at the 5’-ends were incorporated to facilitate nuclease digestion.

### Expression and purification

Cell culture conditions for expression of the HRV 3C, TEV protease (wild type) and protease cleavable proteins, MTD, beta C1 and 100K are described in Table 2.

**Table 2:** Expression conditions used for proteins expression.

Protein Name	Expression construct name	Protein expression/cell culture conditions
TEV protease	pKLD116-V2-TEV	Overnight grown *E*. *coli* BL21-C41 (DE3) [16] Cultures were diluted 1:50 in fresh LB media supplemented with 50 μg/mL ampicillin, were grown to A_600_ 0.5 at 37°C and then expression was induced by the addition of 0.5 mM IPTG for 16 h at 15°C.
HRV 3C protease	pKLD116-V2-HRV 3C	Overnight grown *E*. *coli* Rosetta 2 (DE3) Cultures were diluted 1:50 in fresh LB media supplemented with 50 μg/mL ampicillin, were grown to A_600_ 0.5 at 37°C and then expression was induced by the addition of 0.2 mM IPTG for 16 h at 18°C.
His_8_-MBP-HRV 3C-MTD (or) His_8_-MBP-TEV-MTD	pKLD116-V2-HRV 3C- MTD (or) pKLD116-V2-TEV-MTD	Overnight grown *E*. *coli* Rosetta 2 (DE3) Cultures were diluted 1:50 in fresh LB media supplemented with 50 μg/mL ampicillin, were grown to A_600_ 0.5 at 37°C and then expression was induced by the addition of 1 mM IPTG for 5 h at 22°C.
His_8_-MBP-HRV 3C-betaC1 (or) His_8_-MBP-TEV-beta C1	pKLD116-V2-HRV 3C-betaC1 (or) pKLD116-V2-TEV-betaC1	Overnight grown *E*. *coli* BL21 (DE3) Cultures were diluted 1:50 in fresh LB media supplemented with 50 μg/mL ampicillin, were grown to A_600_ 0.6 at 37°C and then expression was induced by the addition of 1 mM IPTG for 5 h at 37°C.
His_8_-HRV 3C-100K (or) His_8_-TEV-100K	PET28a-HRV 3C-100K (or) pET28a-TEV-100K	Overnight grown *E*. *coli* BL21 (DE3) Cultures were diluted 1:50 in fresh LB media supplemented with 25 μg/mL kanamycin, were grown to A_600_ 0.5–0.6 at 37°C and then expression was induced by the addition of 0.5 or 1 mM IPTG for 4–5 h at 37°C [17].

*E*. *coli* cells containing over-expressed HRV 3C protease were resuspended in 20 mM Tris-HCl pH 8.0 containing 500 mM NaCl, 5 mM imidazole and 4 mM β-mercaptoethanol (β-ME) (buffer A) whereas TEV protease containing cells of *E*. *coli* were resuspended in 20 mM Tris-HCl pH 8.0 containing 500 mM NaCl, 20 mM imidazole and 10% glycerol (buffer B). Resuspended cells were broken by a Cell disruptor (Constant Systems Ltd, UK) at pressure of 20 kpsi and the cell lysate was spun at 12,000×g, 4°C for 30 min to remove unbroken cells, cell debris or inclusion bodies (if any). The supernatant was passed twice through a Nickel-nitrilotriacetate agarose column equilibrated with the respective resuspension buffers and washed with 25 column volume (CV) of their respective wash buffers (buffer A with 25 mM imidazole and buffer B with 20 mM imidazole). Proteins were eluted in their respective buffers A and B with 250 mM and 500 mM imidazole, respectively. Purified HRV 3C and TEV proteases were dialysed against 20 mM Tris-HCl pH 7.5 containing 150 mM NaCl, 50% glycerol, 4 mM β-ME and 5 mM EDTA (storage buffer). The protein concentrations were measured by a Bradford assay [18], aliquoted (4 mg per mL) and stored at -20°C until further use.

*E*. *coli* cells containing overexpressed protease cleavable proteins His_8_-MBP-HRV 3C-MTD, His_8_-MBP-TEV-MTD, His_8_-MBP-HRV 3C-beta C1, His_8_-MBP-TEV-beta C1, His_8_-HRV 3C-100K or His_8_-TEV-100K were resuspended in 20 mM Tris-HCl pH 7.5 containing 300 mM NaCl, 5 mM imidazole and 4 mM β-ME (buffer C). Resuspended cells were broken by a Cell disruptor (Constant Systems Ltd, UK) at pressure of 20 kpsi and the cell lysate was spun at 12,000×g, 4°C for 30 min to remove unbroken cells, cell debris or inclusion bodies (if any). The supernatant was passed twice through a Nickel-nitrilotriacetate agarose column equilibrated with the respective resuspension buffers and washed with 25 column volume (CV) of wash buffers (buffer C with 30 mM imidazole). Proteins were eluted in buffer C containing 150 mM imidazole and 100 mM NaCl. Purified proteins were dialysed against 20 mM Tris-HCl pH 7.5 containing 150 mM NaCl. The protein concentrations were measured by a Bradford assay [18], aliquoted (4 mg per mL) and stored at -20°C until further use.

### Optimization of cleavage conditions for HRV 3C and TEV protease

Protease cleavable proteins (each 50 μg), MTD (His8-MBP-HRV 3C-MTD or His8-MBP-TEV-MTD), beta C1 (His_8_-MBP-HRV 3C-beta C1 or His_8_-MBP-TEV-beta C1) and 100K (His_8_-HRV 3C-100K or His_8_ -TEV-100K) were incubated with HRV 3C or TEV protease (each 2 μg) at a ratio of 50:1 (w/w) in 20 mM Tris-HCl pH 7.5, 150 mM NaCl and 2 mM β-ME in a total volume of 100 μl at 25°C and 4°C. After 1, 2, 4, 8 and 16 hr aliquots were removed from the cleavage reaction, mixed with SDS-PAGE sample buffer (125 mM Tris-HCl pH 6.8 containing 2.5% (w/v) sodium dodecyl sulfate, 0.1% (w/v) ethylenediamine tetraacetate, 12.5% (v/v) glycerol and 0.1% (w/v) bromophenol blue) and stored at -20°C until analyzed. For analysis, 5 μl aliquots of cleavage reaction mixture were loaded in each lane and fusion proteins corresponding to MTD/beta C1 and 100K were resolved using 12% and 10% SDS-PAGE, respectively.

### Analysis of activity of HRV 3C protease in various buffers, additives and detergent solutions

The effect on the activity of HRV 3C protease was investigated at 4°C for the following buffers and additives: (1) frequently used buffers for the elution of polyhistidine-tagged (50 mM Na_2_HPO_4_, 0.3 M NaCl, 300 mM imidazole, pH 8.0), maltose binding protein (MBP) -tagged (20 mM Tris—HCl pH 7.4 containing 200 mM NaCl, 1 mM EDTA, 1 mM DTT and 10 mM maltose), glutathione-s-transferase (GST) -tagged (50 mM Tris—HCl, 10 mM reduced glutathione, pH 8.0) or Strep-tagged (50 mM Tris—HCl, 150 mM NaCl, 1 mM EDTA, 2.5 mM desthiobiotin or biotin, pH 8.0) proteins from the relevant affinity resins; (2) salts (NaCl, KCl, CaCl_2_, MgCl_2_, MgSO_4_, ZnSO_4_); (3) protein stabilizing/solubilizing agents (sorbitol, trehalose, glycerol, arginine and proline); (4) denaturants (sodium dodecyl sulfate, guanidine-HCl and urea); (5) reducing agents (β-ME, dithiothreitol (DTT), tris(2-carboxyethyl) phosphine (TCEP)); and (6) detergents (see Table 3). His8-MBP-HRV 3C-MTD was incubated with HRV 3C protease at a ratio of 1:50 (w/w) at 4°C for 4 hr in 20 mM Tris-HCl pH 7.5 containing 150 mM NaCl and 2 mM β-ME. The reaction was terminated by the addition of SDS-PAGE sample buffer and samples were stored at– 20°C until analyzed. The HRV 3C protease cleavage efficiency was estimated visually from SDS-PAGE gels stained with Coomassie Brilliant Blue.

**Table 3:** Effect of detergents on the activity of the HRV 3C protease activity.

S.No.	Chemical Name	Critical micelle concentration	Activity
1	ANAPOE^®^-80	1% w/v	+++
2	n-Tetradecyl-β-D-maltoside	0.0335 mM	+++
3	ANAPOE^®^-C13E8	0.1% w/v	+++
4	C12E8	0.11 mM	+++
5	ANAPOE^®^-C12E10	1% w/v	+++
6	Sucrose monolaurate	0.3 mM	+++
7	n-Undecyl-b-D-maltoside	0.59 mM	++
8	CYMAL^®^-6	0.56 mM	+++
9	n-Nonyl-b-D-thioglucoside	0.15 mM	++
10	CYMAL^®^ -5	5 mM	++
11	n-Nonyl-β-D-maltoside	6 mM	++
12	C8E4	8 mM	++
13	C-HEGA^®^-11	11.5 mM	++
14	n-Octyl-β-D-glucoside	20 mM	++
15	MEGA-9	25 mM	+
16	2,6-Dimethyl-4-heptyl-β-D-maltopyranoside	27.5 mM	+++
17	C-HEGA^®^-10	35 mM	0
18	HEGA^®^-9	39 mM	+
19	C-HEGA^®^-9	108 mM	0
20	HEGA^®^-8	109 mM	0
21	CYMAL^®^-2	120 mM	0
22	n-Hexyl-β-D-glucopyranoside	250 mM	+
23	NDSB-195	50 mM	+++
24	FOS-Choline^®^-12	1.5 mM	++
25	FOS-Choline^®^-8, fluorinated	2.2 MM	++
26	ZWITTERGENT^®^ 3–12	4 mM	+
27	CHAPS	8 mM	++
28	CHAPSO	8 mM	++
29	n-Decyl-N,N-dimethylglycine	18 mM	0
30	ANAPOE^®^-58	1% w/v	++
31	MEGA -10	7 mM	+
32	CYMAL^®^-4	7.6 mM	++
33	Pluronic^®^ F-68	1% w/v	+
34	HECAMEG^®^	19.5 mM	+
35	Sulfobetaine 3–10	25 mM	++
36	CYMAL^®^-3	34.5 mM	+
37	MEGA-8	79 mM	0
38	NDSB-256	50 mM	+
39	DDMAB	4.3 mM	+++
40	FOS-MEA^®^-10	5.2 mM	+
41	ZWITTERGENT^®^ 3–10	40 mM	+++
42	FOS-Choline^®^-8	114 mM	0
43	LysoFos^™^ Choline 12	0.7 mM	+
44	LysoFos^™^ Choline 10	7 mM	+++
45	Sodium dodecanoylsarcosine	14.4 mM	+
46	ANAPOE^®^-20	1% w/v	0
47	n-Dodecyl-β-D-maltoside	0.17 mM	+++
48	TRITON^®^ X-100	0.9 mM	+++
49	LDAO	2 mM	++

## Results and Discussion

**Fig 1:**
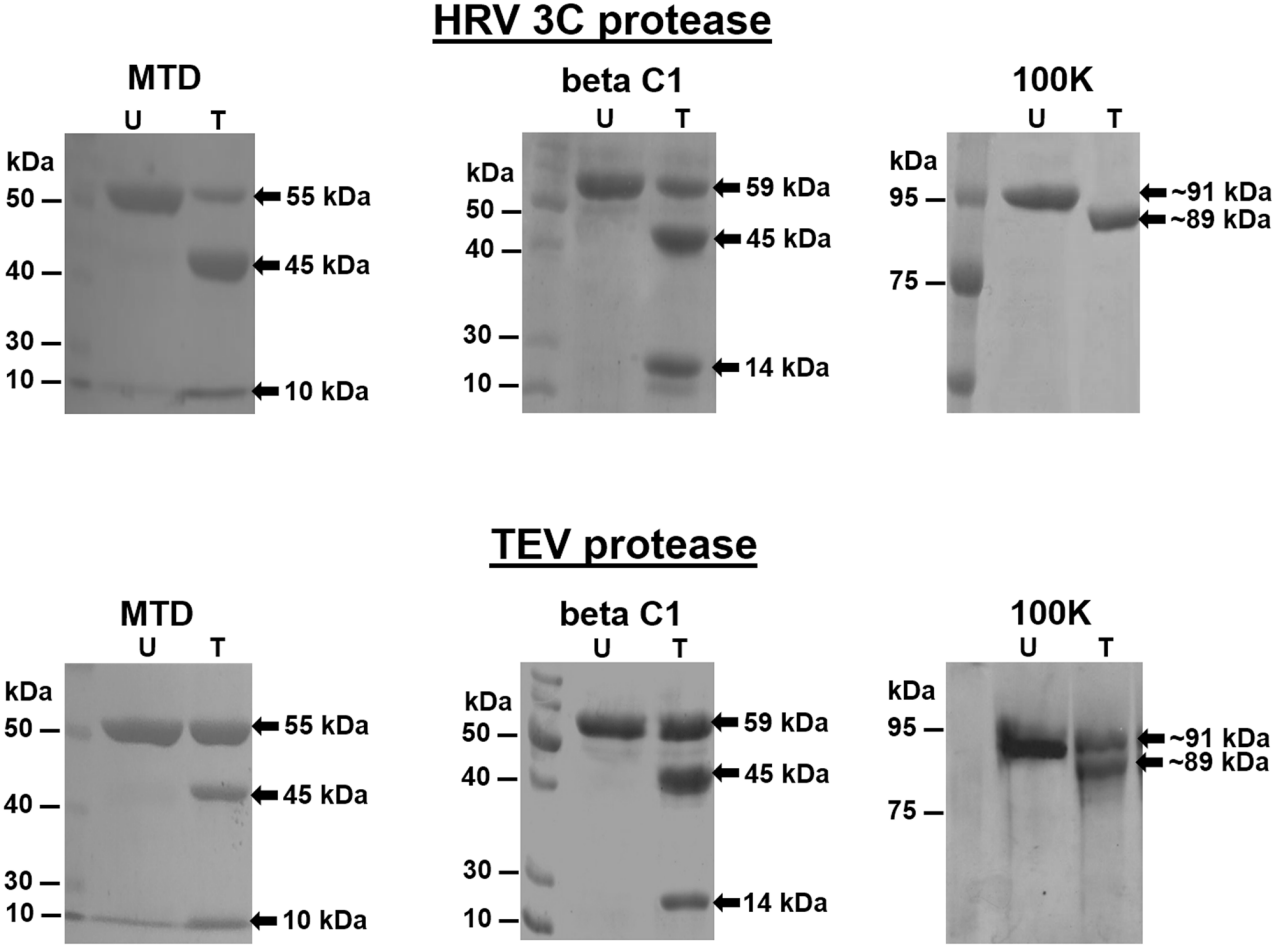
Analysis of activities of the HRV 3C and TEV proteases at 25°C towards their target proteins (His8-MBP-HRV 3C-MTD or His8-MBP-TEV-MTD, His_8_-MBP-HRV 3C-betaC1 or His_8_-MBP-TEV-beta C1 and His_8_-HRV 3C-100K or His_8_-TEV-100K). Respective cleavable fusion proteins were incubated with HRV 3C protease or TEV protease at ratio of 50:1 for 1 hour and analysed by SDS-PAGE. ‘‘U” and ‘‘T” represent the cleavable fusion protein untreated and treated, respectively, with the HRV 3C or TEV protease, Note: in case of cleavage of 100K fusion protein, 2kDa band was not observed because of low %age of polyacrylamide used in SDS-PAGE.

Protease cleavable proteins His_8_-MBP-HRV 3C-MTD or His_8_-MBP-TEV-MTD, His_8_-MBP-HRV 3C-betaC1 or His_8_-MBP-TEV-beta C1 and His_8_-HRV 3C-100K or His_8_-TEV-100K have calculated molecular masses of ~ 55.2 kDa, ~ 59.2 kDa and ~ 91 kDa, respectively. Molecular masses of proteins were calculated using the ExPASy Bioinformatic Portal, http://web.expasy.org/compute_pi/. All proteins were produced by recombinant means as described in materials and methods. Cleavage of the target fusion proteins with the HRV 3C or TEV protease releases two major protein bands of ~ 45 kDa (His_8_-MBP) and ~ 10 kDa (MTD), ~ 45 kDa (His_8_-MBP) and 14 kDa (beta C1), and ~89 kDa (100K) and ~2 kDa (His_8_), respectively (Fig 1). For clarity, we only show the bands corresponding to the uncut fusion proteins and large size cut fragments of the fusion proteins in subsequence SDS-polyacrylamide gels.

**Fig 2:**
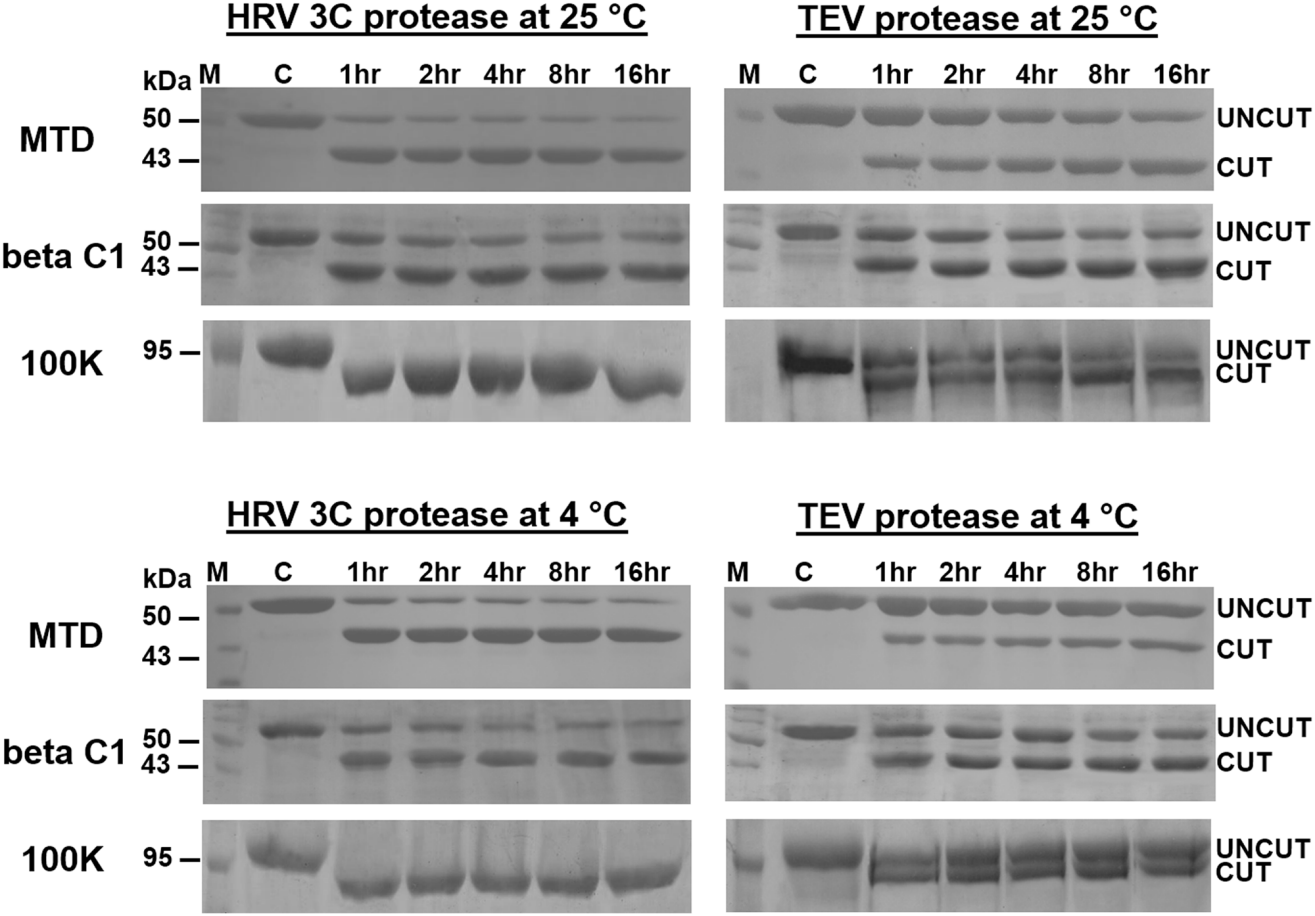
Analysis of activity of the HRV 3C protease and the TEV protease towards their target proteins (His_8_-MBP-HRV 3C-MTD, His_8_-MBP-TEV-MTD, His_8_-MBP-HRV 3C-betaC1, His_8_-MBP-TEV-beta C1, His_8_-HRV 3C-100K and His_8_-TEV-100K) at 25°C and 4°C. The HRV 3C protease or the TEV protease was incubated with respective cleavable fusion protein at ratio of 1:50 and aliquots were taken at the indicated time points and analysed by SDS-PAGE. M denotes the molecular marker while ‘‘C” represents the cleavable fusion protein without any of protease treatment.

We compared the activities of the HRV 3C and TEV proteases towards their three target proteins at 25°C and 4°C. Fig 2 shows that, whilst TEV protease efficiently cleaves the target proteins except His_8_-TEV-100K after prolonged incubation at 25°C, it was inefficient to do so even after 16 hr at 4°C. In contrast, HRV 3C protease efficiently cleaved all the three fusion proteins after 4 hr at both tested temperatures. Since many recombinant proteins require handling at 4°C, starting from protein purification through to downstream processing, the superior cleavage properties of HRV 3C protease over TEV protease demonstrate the potential to use HRV 3C protease for recombinant protein production [19]. In addition, HRV 3C protease should also be the protease of choice at 25°C, as our experiments show that its activity at higher temperatures is also superior to that of TEV protease (Fig 2).

**Fig 3:**
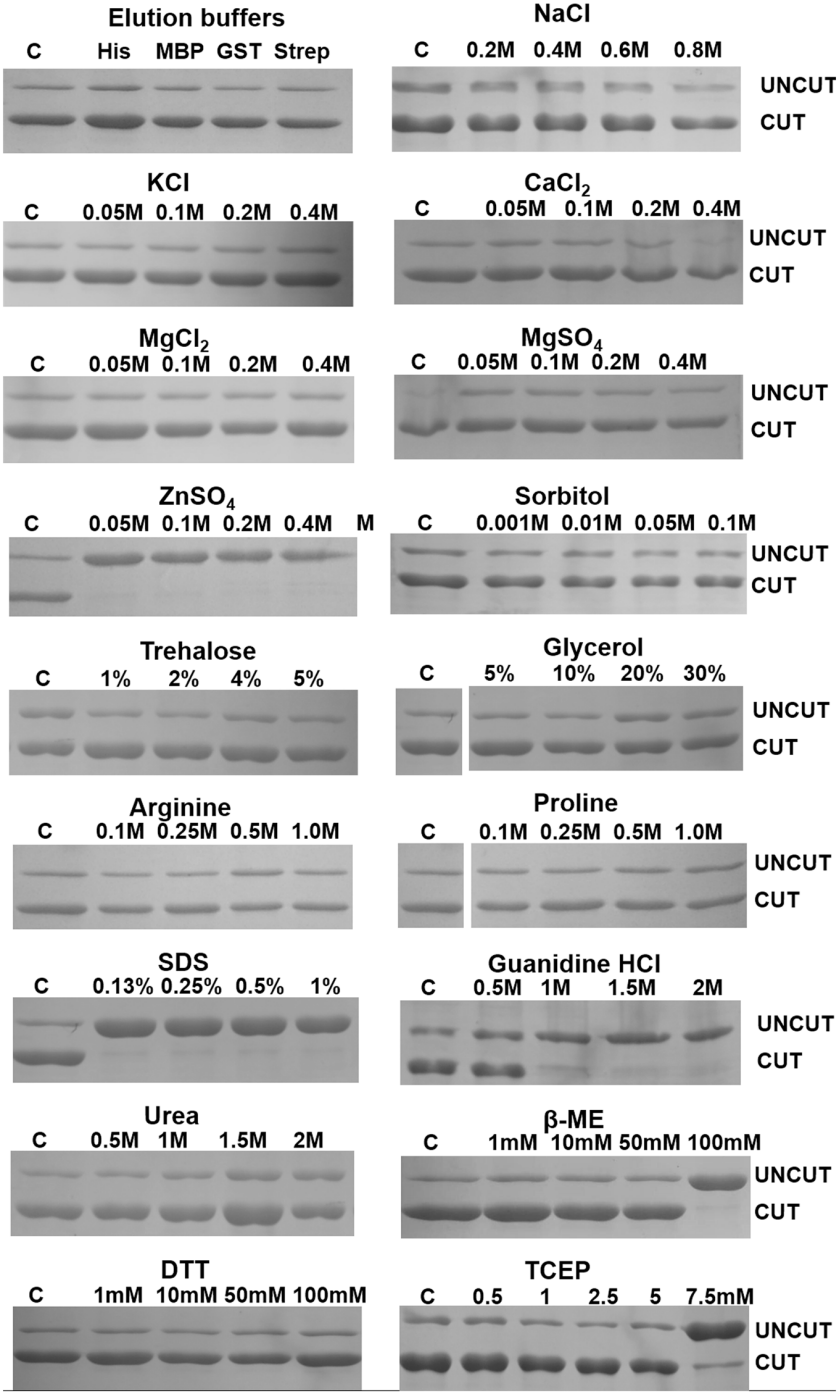
SDS-PAGE showing the HRV 3C protease activity in the presence various buffers, increasing concentrations of variety of salts, additives and detergents commonly used during extraction and purification of proteins. ‘‘C” represents the cleavable fusion protein treated with protease in the absence of any additive.

To further assess the wide applicability of HRV 3C protease, we tested the effect of the buffer composition on the activity. HRV 3C protease cleaved His_8_-MBP-HRV 3C-MTD efficiently upon 4 hr of incubation at 4°C in each of the four standard buffers used for the elution of polyHis-, GST-, MBP- and Strep-tagged fusion proteins [1, 20–23] (Fig 3). This indicates the robustness of the HRV 3C cleavage protein in a variety of buffers; hence eliminating the time consuming process of desalting or dialysis. Furthermore, rapid cleavage by HRV 3C protease limits compromising target protein integrity. The broad cleavage efficiency of HRV 3C protease identifies it as the protease of choice. In cases where the target protease cleavable protein is unstable in the elution buffer (for example, due to a high imidazole concentration in purifications using Ni-NTA technology), exchange into a suitable buffer by dialysis or any other procedure may be done before cleavage is performed.

Salt ions affect the stability, solubility, structure and function of proteins in various ways [11, 19, 24]. One of these mechanisms is the interaction of salt ions with counter-ions on the protein, reducing electrostatic attraction between proteins and thereby enhancing the solubility of the target protein. This suggests that a defined amount of salt in buffers is required for the stability and solubility of proteins. We tested the cleavage of His_8_-MBP-HRV 3C-MTD by the HRV 3C protease in presence of increasing concentrations of NaCl, KCl, CaCl_2_, MgCl_2_, MgSO_4_ and ZnSO_4_. With the exception of ZnSO_4_, none of the salt ions inhibited the activity of the HRV 3C protease at concentrations between 0.05 and 0.4 M (Fig 3).

Additives like sorbitol, trehalose, glycerol, arginine and proline are added during the extraction and purification of proteins to aid solubilization [10]. Fig 3 shows only limited effects upon adding these molecules into the cleavage reaction; hence, their removal is not necessary prior to cleavage. Strong denaturants like SDS at tested concentration of 0.13% and also guanidine-HCl when present above 0.5 M severely inhibited the activity of the HRV 3C protease (Fig 3). However, the HRV 3C protease exhibited optimal activity towards the target protein in the presence of urea up to a concentration of 2 M (Fig 3). Higher urea concentrations might impair HRV 3C protease activity also, however, these were not tested in this study. The reducing agents β-ME, DTT and TCEP are often used to maintain the activity and the stability of target proteins or to prevent protein aggregation. HRV 3C protease activity persisted in the presence of up to 50 mM β-ME, 100 mM DTT and 5 mM TCEP, which are up to or far above typical concentrations used in recombinant protein production (Fig 3)

**Fig 4:**
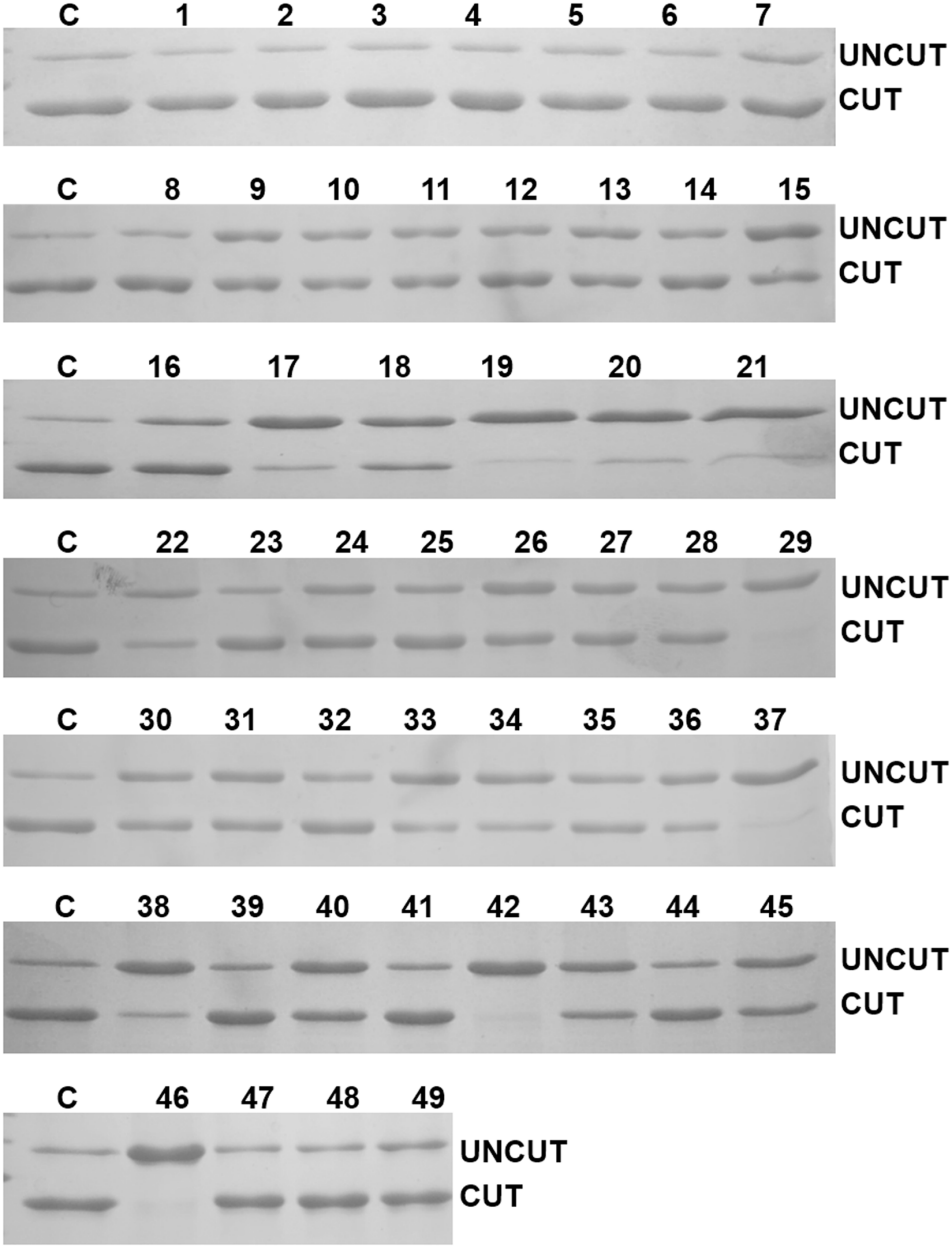
SDS-PAGE analysis of cleavage of His_8_-MBP-HRV 3C-domain by the HRV 3C protease in the presence of various detergents. Each lane is labeled according to detergents serial number as detailed in Table 3. ‘‘C” represents the cleavable fusion protein treated with protease in the absence of any detergent.

Finally, we assessed the activity of the HRV 3C protease in the presence of different detergents at 4°C. These chemicals are used for different purposes, including the extraction of hydrophobic proteins from biological membranes, refolding, crystallization and protein stabilization. We digested His_8_-MBP-HRV 3C-MTD in the presence of various detergents at their critical micelle concentration with HRV 3C protease (Table 3 and Fig 4). Of the forty nine detergents tested, fourteen detergents (ANAPOE^®^-80, n-Tetradecyl-β-D-maltoside, ANAPOE^®^-C13E8, C12E8, ANAPOE^®^-C12E10, Sucrose monolaurate, CYMAL^®^-6, 2,6-Dimethyl-4-heptyl-β-D-maltopyranoside, NDSB-195, DDMAB, ZWITTERGENT^®^ 3–10, LysoFos^™^ Choline 10, n-Dodecyl-β-D-maltoside and TRITON^®^ X-100) did not affect the HRV 3C protease activity and the target protein was efficiently cleaved (~ 80 to 100%) compared to a control reaction in absence of any detergent. In the presence of fifteen detergents (n-Undecyl-β-D-maltoside, n-Nonyl-β-D-thioglucoside, CYMAL^®^-5, n-Nonyl-β-D-maltoside, C8E4, C-HEGA^®^-11, n-Octyl-β-D-glucoside, FOS-Choline^®^-12, FOS-Choline^®^-8 fluorinated, CHAPS, CHAPSO, ANAPOE^®^-58, CYMAL^®^-4, Sulfobetaine 3–10 and LDAO) HRV 3C protease activity was reduced to ~ 50% relative to the control reaction and the activity decreased further, below 50%, relative to the cleavage in the control reaction in the presence of another twelve detergents (MEGA-9, HEGA^®^-9, n-Hexyl-β-D-glucopyranoside, ZWITTERGENT^®^ 3–12, MEGA-10, Pluronic^®^ F-68, HECAMEG^®^, CYMAL^®^-3, NDSB-256, FOS-MEA^®^-10, LysoFos^™^ Choline 12, and Sodium dodecanoylsarcosine). HRV 3C protease activity was completely abolished in the presence of eight detergents (C-HEGA^®^-10, C-HEGA^®^-9, HEGA^®^-8, CYMAL^®^-2, n-Decyl-N,N-dimethylglycine, MEGA-8, FOS-Choline^®^-8 and ANAPOE^®^-20). In 2011, Vergis and Weiner [4] reported a study on the cleavage activity of HRV 3C protease in the presence of a wide range of detergents at 25°C. Although the substrates are different and half of the detergents tested are different, comparison of both datasets for the same detergents show that, in most cases, HRV 3C protease activity is preserved better at 4°C. For example, whereas Vergis and Weiner [4] report little or no cleavage in the presence of FOS-choline-12, HRV 3C protease cleaves over 50% of the target protein at 4°C. In contrast, cleavage is better at 25°C in the presence of C8E4, HECAMEG and n-undecyl-b-D-maltoside [4]. On the basis of structure of detergents, it has been observed that HRV 3C protease is inhibited to less extent by maltoside and glycol ether containing detergents.

The extent to which the HRV 3C protease has cleaved His8-MBP-HRV 3C-domain in presence of various detergents at their critical micelle concentration is shown in Fig 3 and represented as follows ‘+++’ for cleavage ~ 80 to 100%, ‘++’ for ~ 50% and ‘+’ for <50%, ‘0’ for almost no cleavage at all compared to control. Critical micelle concentration of detergents was taken from http://www.sigmaaldrich.com/content/dam/sigma-aldrich/docs/Sigma/Instructions/detergent_selection_table.pdf

We show that HRV 3C protease activity is insensitive to frequently used buffers and additives in recombinant protein production. Importantly, our results demonstrate the superior cleavage properties of HRV 3C protease at 4°C, a temperature of choice for the processing of many proteins, though dataset was comprised of only three fusion proteins. These characteristics make HRV 3C protease the protease of choice for the removal of tags from fusion proteins. This is of relevance for HRV 3C protease to be exploited to enhance the large-scale production of proteins of industrial and pharmaceutical importance.
