# Correction: Activity of the Human Rhinovirus 3C Protease Studied in Various Buffers, Additives and Detergents Solutions for Recombinant Protein Production

**DOI:** 10.1371/journal.pone.0160128

**Published:** 2016-07-21

**Authors:** Raheem Ullah, Majid Ali Shah, Soban Tufail, Fouzia Ismat, Muhammad Imran, Mazhar Iqbal, Osman Mirza, Moazur Rahman

The eighth author’s name is incorrectly spelled. The correct name is: Moazur Rahman.

The footnote for Table 3 appears incorrectly in the Results and Discussion section. Please see the correct Table 3 and footnote here.

**Table 3:** Effect of detergents on the activity of the HRV 3C protease activity.

S.No.	Chemical Name	Critical micelle concentration	Activity
1	ANAPOE^®^-80	1% w/v	+++
2	n-Tetradecyl-β-D-maltoside	0.0335 mM	+++
3	ANAPOE^®^-C13E8	0.1% w/v	+++
4	C12E8	0.11 mM	+++
5	ANAPOE^®^-C12E10	1% w/v	+++
6	Sucrose monolaurate	0.3 mM	+++
7	n-Undecyl-b-D-maltoside	0.59 mM	++
8	CYMAL^®^-6	0.56 mM	+++
9	n-Nonyl-b-D-thioglucoside	0.15 mM	++
10	CYMAL ^®^ -5	5 mM	++
11	n-Nonyl-β-D-maltoside	6 mM	++
12	C8E4	8 mM	++
13	C-HEGA^®^-11	11.5 mM	++
14	n-Octyl-β-D-glucoside	20 mM	++
15	MEGA-9	25 mM	+
16	2,6-Dimethyl-4-heptyl-β-D-maltopyranoside	27.5 mM	+++
17	C-HEGA^®^-10	35 mM	0
18	HEGA^®^-9	39 mM	+
19	C-HEGA^®^-9	108 mM	0
20	HEGA^®^-8	109 mM	0
21	CYMAL^®^-2	120 mM	0
22	n-Hexyl-β-D-glucopyranoside	250 mM	+
23	NDSB-195	50 mM	+++
24	FOS-Choline^®^-12	1.5 mM	++
25	FOS-Choline^®^-8, fluorinated	2.2 MM	++
26	ZWITTERGENT^®^ 3–12	4 mM	+
27	CHAPS	8 mM	++
28	CHAPSO	8 mM	++
29	n-Decyl-N,N-dimethylglycine	18 mM	0
30	ANAPOE^®^-58	1% w/v	++
31	MEGA -10	7 mM	+
32	CYMAL^®^-4	7.6 mM	++
33	Pluronic^®^ F-68	1% w/v	+
34	HECAMEG^®^	19.5 mM	+
35	Sulfobetaine 3–10	25 mM	++
36	CYMAL^®^-3	34.5 mM	+
37	MEGA-8	79 mM	0
38	NDSB-256	50 mM	+
39	DDMAB	4.3 mM	+++
40	FOS-MEA^®^-10	5.2 mM	+
41	ZWITTERGENT^®^ 3–10	40 mM	+++
42	FOS-Choline^®^-8	114 mM	0
43	LysoFos^™^ Choline 12	0.7 mM	+
44	LysoFos^™^ Choline 10	7 mM	+++
45	Sodium dodecanoylsarcosine	14.4 mM	+
46	ANAPOE^®^-20	1% w/v	0
47	n-Dodecyl-β-D-maltoside	0.17 mM	+++
48	TRITON^®^ X-100	0.9 mM	+++
49	LDAO	2 mM	++

The extent to which the HRV 3C protease has cleaved His8-MBP-HRV 3C-domain in presence of various detergents at their critical micelle concentration is shown in Fig 3 and represented as follows ‘+++’ for cleavage ~ 80 to 100%, ‘++’ for ~ 50% and ‘+’ for <50%, ‘0’ for almost no cleavage at all compared to control. Critical micelle concentration of detergents was taken from http://www.sigmaaldrich.com/content/dam/sigma-aldrich/docs/Sigma/Instructions/detergent_selection_table
